# Correlates of Normal and Abnormal General Movements in Infancy and Long-Term Neurodevelopment of Preterm Infants: Insights from Functional Connectivity Studies at Term Equivalence

**DOI:** 10.3390/jcm9030834

**Published:** 2020-03-19

**Authors:** Colleen Peyton, Christa Einspieler, Toril Fjørtoft, Lars Adde, Michael D. Schreiber, Alexander Drobyshevsky, Jeremy D. Marks

**Affiliations:** 1 Department of Pediatrics, University of Chicago, Chicago, IL 60422, USA; 2 Department of Physical Therapy and Human Movement Science and the Department of Pediatrics, Northwestern University, Chicago, IL 60611, USA; 3 Research Unit IDN, Interdisciplinary Developmental Neuroscience, Division of Phoniatrics, Medical University of Graz, Graz 8036, Austria; christa.einspieler@medunigraz.at; 4 Department of Clinical and Molecular Medicine, Faculty of Medicine and Health Sciences, Norwegian University of Science and Technology, 7491 Trondheim, Norway; Toril.Fjortoft@stolav.no (T.F.); Lars.adde@ntnu.no (L.A.); 5 Clinics of Clinical Services, St. Olavs Hospital, Trondheim University Hospital, 7006 Trondheim, Norway; 6 Department of Pediatrics, NorthShore University HealthSystem, Evanston, IL 60201, USA; oldrobys@gmail.com; 7 Department of Neurology, University of Chicago, Chicago, IL 60422, USA

**Keywords:** general movements, perinatal brain injury, functional brain connectivity, preterm infant, Bayley scale, fidgety movements

## Abstract

Preterm infants born before 32 weeks gestation have increased risks for neurodevelopmental impairment at two years of age. How brain function differs between preterm infants with normal or impaired development is unknown. However, abnormal spontaneous motor behavior at 12–15 weeks post-term age is associated with neurodevelopmental impairment. We imaged brain blood oxygen level-dependent signals at term-equivalent age in 62 infants born at <32 weeks gestation and explored whether resting state functional connectivity (rsFC) differed with performances on the General Movement Assessment (GMA) at 12–15 weeks, and Bayley III scores at two years of corrected age. Infants with aberrant general movements exhibited decreased rsFC between the basal ganglia and regions in parietal and frontotemporal lobes. Infants with normal Bayley III cognitive scores exhibited increased rsFC between the basal ganglia and association cortices in parietal and occipital lobes compared with cognitively impaired children. Infants with normal motor scores exhibited increased rsFC between the basal ganglia and visual cortices, compared with children with motor impairment. Thus, the presence of abnormal general movements is associated with region-specific differences in rsFC at term. The association of abnormal long-term neurodevelopmental outcomes with decreased rsFC between basal ganglia and sub-score specific cortical regions may provide biomarkers of neurodevelopmental trajectory and outcome.

## 1. Introduction

Preterm infants born before 32 weeks of gestation are at increased risk of motor, cognitive, and language impairment compared with infants born at term. Cerebral palsy, one of these motor deficits, is not often diagnosed until 12–24 months of corrected age. However, preterm infants can be identified between 10 and 15 weeks post term age as being at high risk for developing cerebral palsy through the assessment of spontaneous movements, called fidgety movements (FMs) [1,2]. FMs are a pattern of continuous, small-amplitude movements of the neck, trunk, and limbs that appear during wakefulness and disappear in sleep or with agitation [3]. Absent or abnormal FMs, as measured by a General Movement (GM) assessment [4], are early indicators of subsequent cerebral palsy. We previously reported that, compared with infants with infants with normal FMs, infants with aberrant FMs at 12–15 weeks post conceptional age exhibit specific white matter microstructure abnormalities at term-equivalent age. Infants with aberrant FMs exhibit lower fractional anisotropy in multiple regions, including the corpus callosum genu and splenium, superior and inferior longitudinal and fronto-occipital fasciculi, the anterior limb of the internal capsule, corona radiata, and optic radiations [5].

In addition to FMs, infants exhibit a rich repertoire of spontaneous behaviors and exhibit other, well-described movement components. These additional behaviors are captured by the motor optimality score (MOS) [3]. In addition to FMs, the MOS scores infants on four other axes: motor repertoire, movement quality, posture, and movement character. While children born preterm with normal FMs are unlikely to develop cerebral palsy [1], infants with normal FMs but without smooth and fluent (i.e., normal) movement character are at increased risk for other important developmental abnormalities at as late as 10 years of age. Abnormalities include reduced cerebral white matter volumes [6], decreased cognition [7], poorer performance on expressive language tests [7], and the development of complex minor neurologic dysfunction [8]. Decreased MOS performance has also been associated with worse cognitive outcomes in very low birth weight (<1500 g) infants at 10 years of age [9] and with adverse neurological outcome in preterm infants at 7–11 years of age [10]. 

Why abnormalities in motor behavior in infants are associated with cognitive and language outcomes in middle childhood is unclear. In infants, functional Magnetic Resonance Imaging (fMRI) studies of resting state brain activity provide data that are independent of state of arousal and are straightforward to implement. Thus, studies employing resting state fMRI in cohorts of premature infants at different post-conceptional ages have consistently identified groups of functionally connected brain regions that develop during gestation, including the default mode network [11] and visual, auditory, somatosensory, motor, and fronto-parietal regions [12,13,14]. It has been suggested that differences in resting state connectivity (rsFC) between normal and injured preterm infants may become useful as potential biomarkers of subsequent long-term cognitive and motor neurodevelopmental outcomes [14]. However, studies linking functional connectivity in infancy and long term neurodevelopmental outcomes are lacking.

In this study, we hypothesized that, in very low birthweight preterm infants at term-corrected age, there is a significant relationship between resting state functional connectivity between brain regions and subsequent performance on movement assessments at three months post-term age. We also hypothesized that, in these infants, there are significant differences at term-corrected age in the resting state functional connectivity between the brain regions of infants who go on to have normal, long-term, cognitive, language, and motor neurodevelopmental outcomes and those who have adverse neurodevelopmental outcomes. Accordingly, we performed unbiased analyses of resting state functional connectivity in preterm infants between all possible pairs of anatomically defined brain regions of interest. As we and others have previously found that abnormalities in motor behavior at 12–15 weeks post term age are associated with long-term neurodevelopmental outcomes [5], differences in the regional rsFC at term may identify the functional systems driving these infant motor behaviors. 

## 2. Materials and Methods

### 2.1. Participants

This study reported on a previously described patient population [15]. Briefly, infants born at ≤31 weeks gestational age and a birth weight of ≤1500 g, who required oxygen at birth, were recruited prospectively between July 2011 and March 2013 from the University of Chicago Comer Children’s Hospital neonatal intensive care unit. Infants with congenital malformations, genetic syndromes, or respiratory distress syndrome that was anticipated to be fatal were excluded from the study. Informed consent was obtained from each infant’s parent according to the Declaration of Helsinki. The study was approved by the university’s institutional review board. 

### 2.2. General Movement Assessment

General movements were assessed with video recordings and a standardized observation system, with the baby in a state of active wakefulness. Recordings were made at 10–15 weeks post term age. Two raters who were certified in the performance of the standardized Prechtl general movement assessment [3] and unaware of the imaging and outcome data analyzed the video recordings to score fidgety movements. As we did previously [5], we classified FMs as either normal or aberrant. FMs were classified as aberrant if abnormal (exaggerated with respect to speed and amplitude), sporadic (interspersed with long pauses), or absent [5]. If the two raters disagreed on classification, a third rater was used.

We next performed the standardized assessment of motor repertoire [3] to produce a motor optimality score [3,16]. In addition to scoring the presence and quality of FMs, this assessment scores movements along four other axes: (1) the number of age-appropriate movements (motor repertoire), (2) the number of pre-specified normal and abnormal movement patterns (movement quality), (3) the ratio of normal to abnormal limb and trunk tonic postures and the presence of abnormal movement patterns (posture), and (4) whether movements are normally smooth and fluent or have one or more abnormal well-described characters, including jerky, monotonous, tremulous, or stiff movements (movement character). To determine the roles played by the group of non-FM behaviors quantified by the assessment of motor repertoire, we subtracted the fidgety movement score from the MOS to obtain a new score: the motor repertoire score. 

### 2.3. MR Imaging

MRI scans were performed at term-equivalent age. Infants were fed an hour prior to the scan and gently restrained, without sedation, with a MedVac immobilization bag (CFI Medical Solutions Inc., Fenton, MI, USA) [17]. Pulse oximetry was used to monitor heart rate and oxygenation throughout the study. Standard hearing protection was applied. Magnetic resonance imaging was performed on a 3 T Philips MRI scanner (Achieva, Best, the Netherlands) with a standard head 8-channel Sensitivity Encoding (SENSE) MRI coil array, designed for adult head imaging with high signal-to-noise ratio and optimum uniformity. The acquisition schema was as follows: 

(1) 3D T1-weighted turbo field echo (Field of View (FOV) 192 × 144 × 124 mm): 1-mm isotropic spatial resolution, inversion time (TI)/repetition time (TR)/echo time (TE) = 1100/8.0/2.9 ms, number of excitations (NEX)=1, turbo field echo factor 144, and a scan duration of 5 min 35 s; (2) resting state blood oxygen level-dependent (BOLD) echo planar imaging: axial slice orientation, 2.5 × 2.5 mm in-plane spatial resolution, 22 slices 3.25 mm thick, FOV 160 × 160 mm, matrix 64 × 63, TR/TE = 1500/28 ms, NEX = 1, SENSE 1.5, 256 volumes, and a scan duration of 6 min 32 s.

### 2.4. Functional MRI Analysis

We employed resting state BOLD imaging to quantify the functional connectivity between brain regions. The resting state functional data were realigned to correct for head motion, co-registered to the T-2 weighted structural image, and normalized to a neonatal automated anatomical labeled atlas space [18] using SPM8 (http://www.fil.ion.ucl.uk/spm). Normalized images were smoothed with an isotropic Gaussian kernel of 5 mm full width half maximum. Individual subjects’ grey and white matter masks, obtained after neonatal brain segmentation as described above, were also normalized to the neonatal atlas space. 

The analysis of resting-state functional data was done in each subject with the CONN toolbox, version 17c [19]. Motion-corrupted scans were scrubbed with the Artifact Detection Tool in the CONN toolbox with settings of global signal Z-value threshold = 5. Subject motion—the maximum voxel displacement resulting from the combined effect of the individual translational (x,y,z) and rotational (pitch, roll, yaw) displacements—was calculated by ART on a scan-by-scan basis. Scans with a motion threshold greater than 0.9 mm were censored and used as nuisance regressors during the denoising process. The minimum number of scans for a subject to be included for analysis was set to 150, providing a minimum of about four minutes of BOLD data [13]. The average number of censored scans excluded from analysis was 21.0 (range: 0–81). There was no significant difference in the number of censored scans per study between the normal and aberrant groups (Normal: 20.5 ± 9.0; Aberrant: 21.1 ± 2.9, *p* = 0.93). 

The realignment parameters of the individual functional image series and motion scrubbing output were entered as first level analysis covariates. The temporal preprocessing of functional time series included band pass 0.008–0.09 Hz filtering, de-trending, and the regression of confounders from the single-subject data: (i) the six realignment parameters computed during image preprocessing, (ii) the time series of the averaged cerebrospinal fluid, and (iii) the averaged white matter signal. The latter time courses were extracted with individual subject segmentation masks, derived as described above. 

For each infant, a region of interest (ROI)-to-ROI functional connectivity analysis was performed with an ROI definition from a neonatal brain atlas [18], which contained anatomical labels for 90 cortical and sub-cortical regions. The average temporal BOLD signal for each ROI was extracted, and bivariate correlations (Fisher Z-transformed correlation coefficients) were computed as a measure of functional connectivity strength between each ROI. 

### 2.5. Assessment of Neurodevelopmental Outcomes

At 18–24 months of corrected age cognitive, language and motor outcomes were assessed with the Bayley Scales of Infant and Toddler Development, Third Edition (Bayley III) [20]. The assessments were performed by two experienced testers who were unaware of the brain MRI findings. Because the Bayley III scores significantly underestimate cognitive and language delay by approximately 11 points [21,22,23], we designated infants having scores ≤85 as having adverse neurodevelopmental outcomes. 

### 2.6. Analysis of Functional Connectivity 

In the group analyses of BOLD signals, connectivity matrices from all subjects were entered into general linear models. The models consisted of explanatory variables of interest that indicated the presence or absence of specific general movement characteristics (e.g., normal vs. aberrant FMs) or continuous variables (e.g., MOSs). In all models, the post-conceptional ages at birth and at MRI were entered as confounding variables. Social and medical risk variables were not included in the model. Region-by-region differences in functional connectivity between every ROI and every other ROI between normal and aberrant FM groups and between normal and adverse Bayley III sub-scores were tested, as were differences in functional connectivity as a function of the MOS.

The patterns of functional connectivity between normal and aberrant fidgety movement groups were tested using the main effects of explanatory variables or appropriate contrasts (i.e., normal fidgety > aberrant fidgety), as described in the results. No a priori hypotheses for specific ROI-to-ROI connectivity were postulated. 

Statistical results were corrected for multiple comparisons with the false discovery rate (FDR) method [19,24] and subjected to a threshold at *p*_FDR_ < 0.05. A seed-level correction threshold was also used. 

### 2.7. Statistical Analysis

To compare the ability of the MOS with that of FM to account for Bayley III scores, we employed multiple linear regression with FM only (Model 1) and FM with non-FM components of the MOS (Model 2) as explanatory variables for the cognitive, language and motor subscales of the Bayley III test. The best subset regression procedure was performed to derive a set of clinical, morbidity, and demographic variables (listed in Table 1) that best explained the variability in the MOSs. Analyses were performed with Minitab 16 (State College, Pa). The mean values of continuous variables between groups were compared with the Student’s t-test. Data are presented as mean ± standard error of mean.

## 3. Results

One hundred and twenty-three infants (Table 1) underwent a general movement assessment and an assessment of motor repertoire at 10–15 weeks post-term age. Of these infants, 107 (87%) also underwent Bayley III testing at about two years of age. The median adjusted age at the time of Bayley III assessment was 24 months (25th percentile: 23.0 months; 75th percentile: 26.25 months). 

### 3.1. The Motor Optimality Score Accounts for a Greater Proportion of the Variance in Bayley III Scores than the Fidgety Movements Score Alone

In contrast to FM assessment, which classifies infants as normal or not, the MOS adds a continuous gradation of motor abnormalities, using components unrelated to FM. Accordingly, we first asked whether the MOS accounts for a greater portion of the variance in long-term neurodevelopmental outcomes as measured by Bayley III subscores than by FM assessment alone. 

For each Bayley III subscore, we compared two linear regression models. For the first model (Model 1), we determined the contribution of the FM scores (normal vs. aberrant) to each Bayley III sub-score. In the second model (Model 2), we divided the MOS into FM and motor repertoire (MR) components (see Methods) and used multiple regression to determine the contributions of these scores to Bayley III sub-scores. For each of the cognitive, language, and motor Bayley III sub-scores, the inclusion of the MR score in the model increased the proportion of the variance that was accounted for by the model (R-squared, Table 2). In fact, for cognitive and language scores, adding the MR score doubled the proportion of variance that was accounted for by the model (Table 2). Thus, the MOS provides a continuous measure of motor function that accounts for a greater proportion of the variance of all Bayley III sub-scores than the binary FM analysis alone. 

To understand the relationships between clinical characteristics known to affect long-term neurodevelopmental outcomes and the MOS, we performed best subset regression of these variables to identify the model with the fewest independent variables that best accounted for the variance in the data. For the MOS, this model identified birth weight, the combined incidence of severe intraventricular hemorrhage (IVH; Grade III or IV), and periventricular leukomalacia (PVL; adjusted R^2^ 0.20; F_(4,115)_=8.34, *p* < 0.001). We then used multiple linear regression to estimate coefficients and *p* values of these variables (birth weight: *p* < 0.001; severe IVH or PVL): *p* = 0.007). For MR scores, best subset analysis identified severe IVH or PVL and, surprisingly, bronchopulmonary dysplasia (BPD) at discharge as accounting for the most variance with the fewest independent variables (adjusted R^2^ 0.16, F_(4,115)_=8.56, *p* < 0.001; IVH/PVL: *p* = 0.006; BPD: *p* = 0001). Notably, birthweight did not account for a significant portion of the variance (*p* = 0.23).

### 3.2. Functional Connectivity Studies

**Figure 1:**
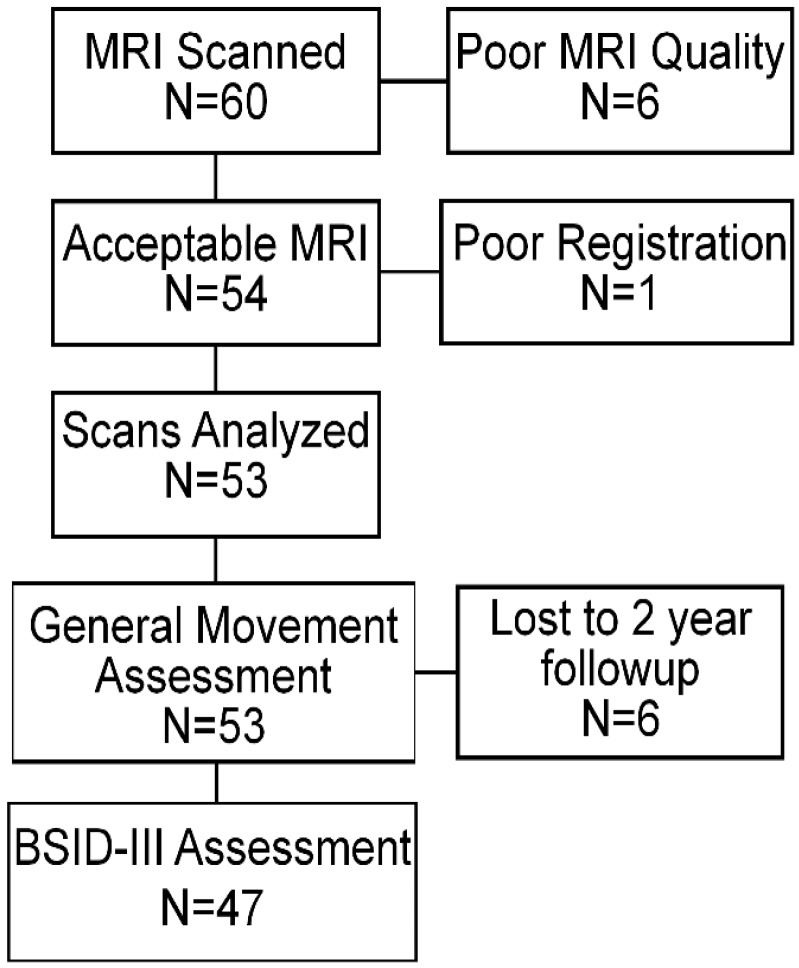
Subject flow through the study.

We next asked whether infants with normal motor behavior at 12–15 weeks post-conceptional age exhibit different patterns of functional connectivity at term-equivalent age compared with infants with abnormal motor behavior. Accordingly, we restricted our cohort to include only those infants who were studied with fMRI (*N* = 60). The parents of study participants were approached for scan consent in the order in which the subject approached term-equivalent age. Patient loss through the study is presented in Figure 1. Of the 60 research scans, 53 were of acceptable quality. The 53 infants with acceptable scans did not differ significantly from the infants in the cohort who were not scanned in terms of clinical characteristics that have been associated with increased incidences of abnormal neurodevelopmental outcomes (Table 3). Accordingly, to perform this exploratory study, we compared resting state functional connectivity between each ROI with every other ROI, and then we conservatively corrected these results for multiple comparisons using the false discovery rate (FDR).

**Figure 2:**
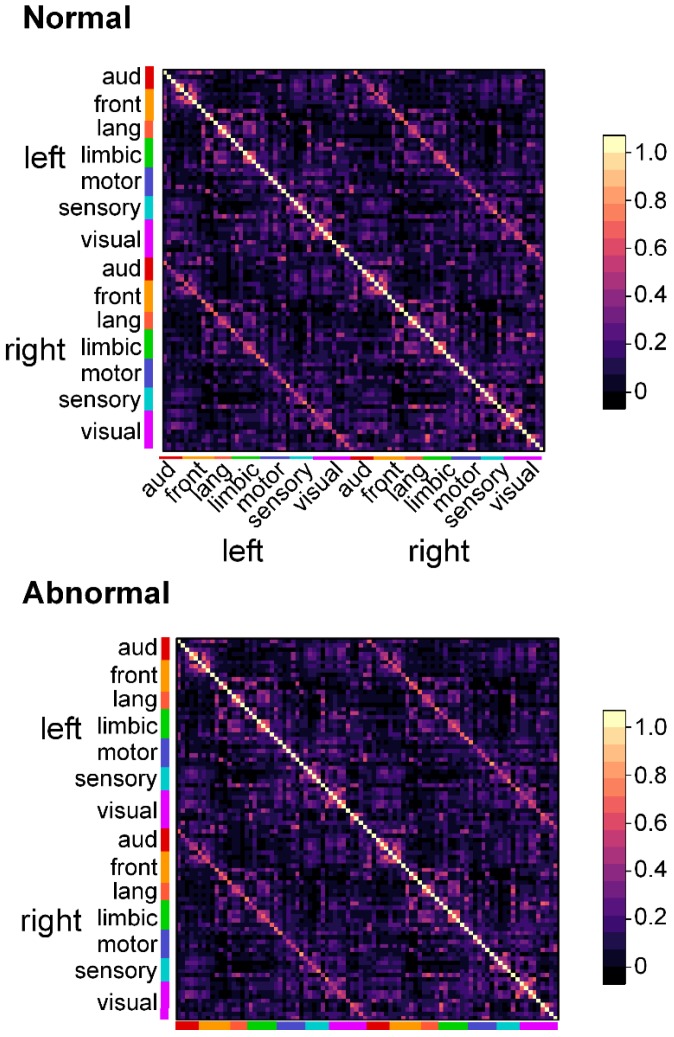
Matrices of mean correlation coefficients between each of the 90 brain regions evaluated in infants with normal and aberrant fidgety movements. Scale bars link the color of each matrix element to the mean correlation coefficient between each region pair.

First, we visualized matrices of the mean correlation coefficients within infants of infants with normal and aberrant FM as heat maps. A visual examination of these plots revealed no gross differences between the groups (Figure 2).

#### 3.2.1. Infants with Normal FMs Demonstrate Greater Functional Connectivity than do Infants with Aberrant FMs

We next identified brain region pairs that differ in resting state connectivity between infants with normal and aberrant FMs, and we compared these results with the differential connectivity that is seen as a function of MOS performance. In this section, as in the sections that follow, all point estimates of the mean temporal BOLD signal correlation coefficients between brain region pairs are reported as Fisher-transformed Z-scores ± one standard error of the mean (SEM).

Of the 53 infants analyzed, 12 infants demonstrated aberrant FMs. These infants had a significantly lower mean birth weight compared with the remainder with normal fidgety movements (Table 4). These infants were also discharged from hospital closer to term corrected age, so that the mean age at MRI scan was significantly greater compared with infants with normal FMs (Table 4). 

**Figure 3:**
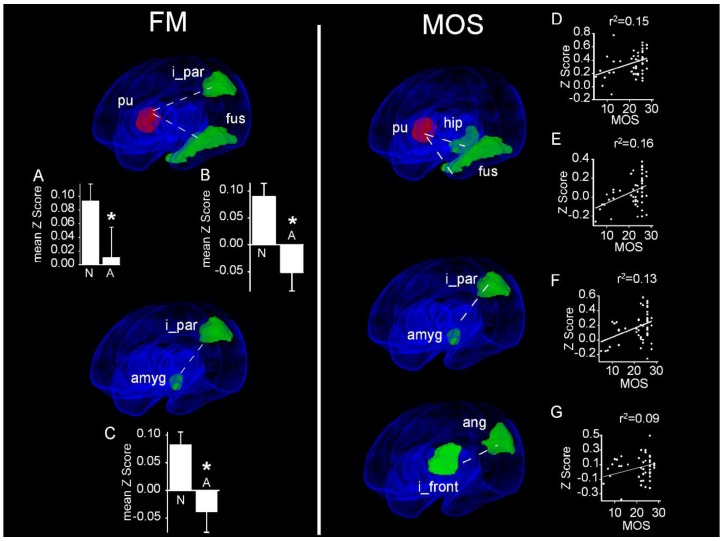
Altered resting state functional connectivity in infants as a function of spontaneous movement. Obliquely projected 3D reconstructions of MRI images of cerebral grey matter and identified parcellated regions derived from a neonatal atlas. FM: Brain regions pairs demonstrating an increased resting state functional connectivity (rsFC) in infants with normal FMs compared with those having aberrant FMs. Insets: Mean Fisher transformed Z-scores ± 1 SEM. (**A**) Putamen and inferior parietal cortex; (**B**) Putamen and fusiform gyrus. (**C**) Amygdala and inferior parietal cortex. MOS (motor optimality score): Brain region pairs demonstrating statistically significant main effects of the MOS on rsFC. Insets: mean Fisher-transformed Z-scores of correlation coefficients. (**D**) Putamen and hippocampus. (**E**) Putamen and fusiform gyrus. (**F**) Inferior parietal cortex and amygdala. (**G**) Inferior frontal gyrus and angular gyrus. Dashed white lines depict connectivity relationships. N: Normal; A: Abnormal. * *P*_FDR_ < 0.05.

A comparison of the fMRI scans of infants with aberrant FMs to those of infants with normal FMs revealed that infants with aberrant FMs exhibited a significantly decreased functional connectivity between the right putamen and both the contralateral inferior parietal cortex (Aberrant: 0.01 ± 0.04 vs. Normal 0.09 ± 0.02, *P*_FDR_ = 0.049) and contralateral fusiform cortex (Aberrant: −0.05 ± 0.03 vs. Normal 0.09 ± 0.02, *P*_FDR_ = 0.046; Figure 3, FM, top). This same inferior parietal cortex also exhibited a decreased functional connectivity with the ipsilateral amygdala (Aberrant: −0.04 ± 0.04 vs. Normal 0.08 ± 0.02, *P*_FDR_ = 0.049; Figure 3 FM, bottom). Importantly, no region pairs in infants with aberrant FMs demonstrated a greater functional connectivity than infants with normal FMs. These data suggest that infants with aberrant FMs have a decreased connectivity between cortical regions integrating visual, somatosensory, and auditory information with deep gray nuclei. 

#### 3.2.2. The Motor Optimality Score Reveals More Functional Connectivity Differences than does the Binary FM Score

In contrast to our FM scoring, which classifies children’s motor performance as normal/aberrant, the motor optimality score is a continuous measure (from 5 to 28 points). We analyzed functional connectivity as a function of the MOS to identify functionally connected regions whose strength of connectivity varied as a function of motor performance from low to high. 

We found significant positive correlations between the MOS and the strength of functional connectivity between multiple region pairs (Figure 3, MOS). Notably, the MOS analysis identified many of the same functionally connected region pairs whose functional connectivity differed significantly between the patient populations using FM scoring. Moreover, this analysis identified more pairs of functionally connected regions than analysis of FM scores alone. Thus, the degree of functional connectivity between the right putamen and contralateral fusiform gyrus was linearly related to the MOS (r^2^ = 0.15, *P*_FDR_ = 0.039). Additional MOS-associated connectivity was identified between the right putamen and the contralateral hippocampus (r^2^ = 0.16, *P*_FDR_ = 0.005). Similar to our FM analysis, connectivity between the left amygdala and ipsilateral inferior parietal cortex was again identified, the strength of which was positively associated with MOSs (r^2^ = 0.13, *P*_FDR_ = 0.028). Finally, MOS-associated functional connectivity—not seen when FM was used as the comparator—was observed between the left angular gyrus and the ipsilateral inferior frontal gyrus (r^2^ = 0.09, *P*_FDR_ = 0.046). Importantly, similar to our FM analysis, we found no region pairs between which functional connectivity strength decreased as a function of the MOS.

### 3.3. Correlates of Movement Character and Functional Connectivity

To better define the brain regions potentially involved in abnormal movement characters, we asked whether infants with different abnormalities of movement character demonstrated differential rsFC values between brain regions. To control for differences in rsFC related to having aberrant FMs, we included only infants with normal FMs (*N* = 43). We compared infants with normal movement character (*N* = 9) with infants classified as having abnormal—monotonous (N=21), tremulous (*N* = 11), stiff (*N* = 14), or jerky (*N* = 22)—movement characters.

#### 3.3.1. Monotonous Movement Character is Associated with Altered Functional Connectivity between Primary Somatosensory Cortex and Visuospatial Regions

**Figure 4:**
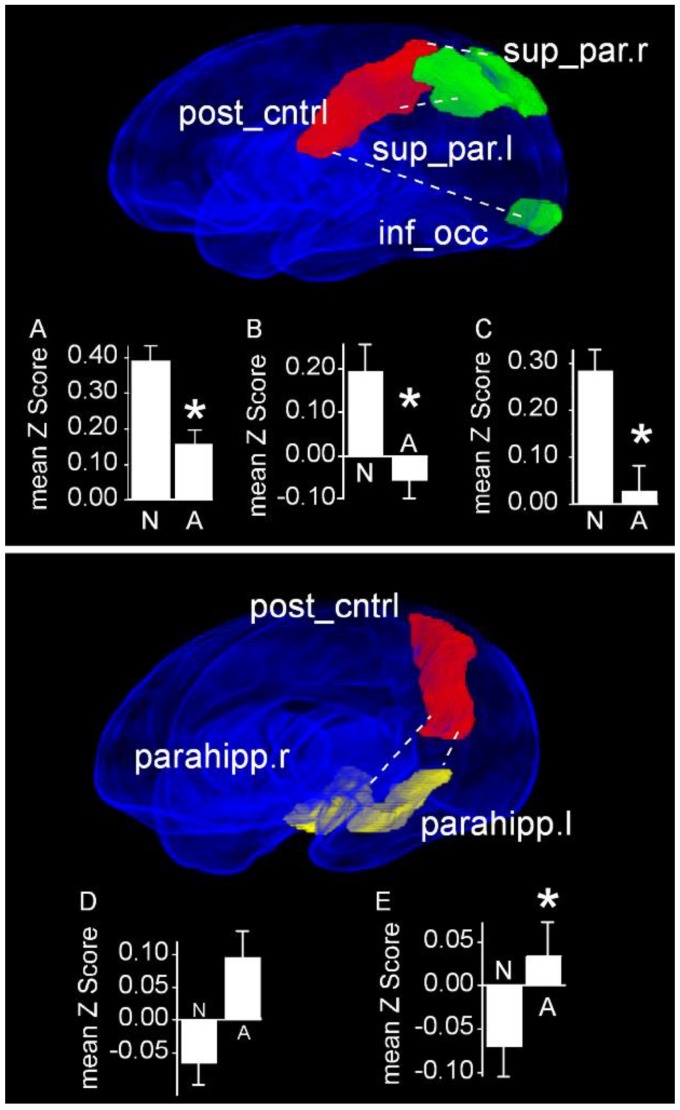
Region pairs exhibiting increased and decreased functional connectivities in infants with normal movement character compared with infants with monotonous movement character. Obliquely projected 3D reconstructions of MRI images of cerebral grey matter and identified parcellated regions derived from a neonatal atlas. Top: The left postcentral gyrus exhibits an increased rsFC with each of the ipsilateral and contralateral superior parietal cortices and the ipsilateral inferior occipital cortex. Bottom: The left postcentral gyrus exhibits a decreased rsFC with each of the ipsilateral and contralateral parahippocampal gyri. Dashed white lines depict connectivity relationships. Insets: Fisher-transformed Z-scores ± 1 SEM. (**A**) Postcentral gyrus and the right superior parietal cortex. (**B**) Postcentral gyrus and the left right superior parietal cortex. (**C**) Postcentral gyrus and inferior occipital cortex. (**D**) Postcentral gyrus and right parahippocampal gyrus. (**E**) Postcentral gyrus and left parahippocampal gyrus. N: Normal; A: Abnormal. * *P*_FDR_ < 0.05.

Twenty-one infants with normal FMs had monotonous movement character. When we compared infants having normal movement character (*N* = 9) with this group, we observed differential functional connectivities between the left postcentral gyrus and several cortical and sub-cortical structures (Figure 4). Specifically, infants with normal movement character exhibited an increased rsFC between the left postcentral gyrus and each of the ipsilateral superior parietal cortex (Normal: 0.39 ± 0.05 vs. Monotonous: 0.15 ± 0.04; *P*_FDR_ = 0.024), the contralateral superior parietal cortex (Normal: 0.19 ± 0.06 vs. Monotonous: −0.06 ± 0.04; *P*_FDR_ = 0.032), and the ipsilateral inferior occipital cortex (Normal: 0.28 ± 0.04 vs. Monotonous: 0.03 ± 0.05; *P*_FDR_ = 0.024; Figure 4, top). Additionally, these infants exhibited a decreased rsFC between the left postcentral gyrus and both the ipsilateral (Normal: −0.07 ± 0.03 vs. Monotonous: 0.12 ± 0.04; *P*_FDR_ = 0.024) and contralateral (Normal: −0.07 ± 0.03 vs. Monotonous: 0.10 ± 0.04; *P*_FDR_ = 0.044) parahippocampal gyrus, compared with infants with monotonous movement character (Figure 4, bottom). 

#### 3.3.2. Stiff Movement Character is Associated with Decreased Connectivity between Visual Cortex and Cingulate Cortices 

**Figure 5:**
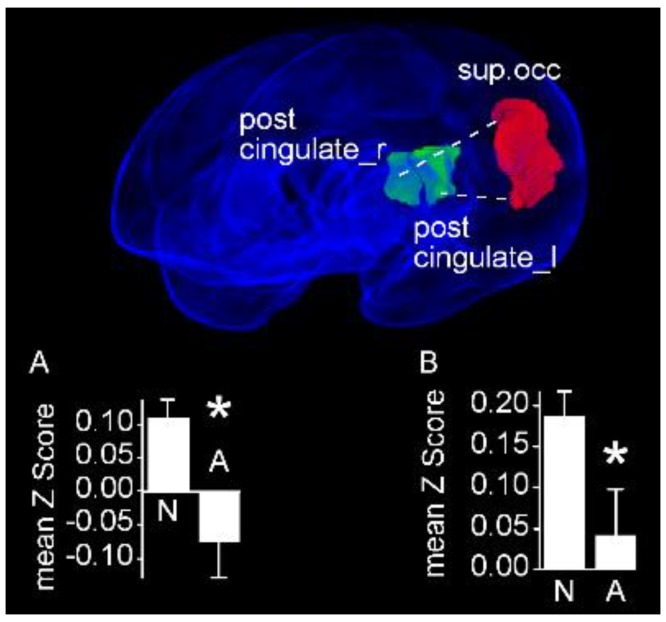
Region pairs exhibiting increased and decreased functional connectivities in infants with normal movement character compared with infants with stiff movement character. Obliquely projected 3D reconstructions of MRI images of cerebral grey matter and identified parcellated regions derived from a neonatal atlas. The left superior occipital cortex exhibits an increased rsFC with both posterior cingulate gyri. Dashed white lines depict connectivity relationships. Insets: Fisher-transformed Z-scores ± 1 SEM. (**A**) Left superior occipital cortex and right posterior cingulate cortex. (**B**) Left superior occipital cortex and left posterior cingulate cortex. N: Normal; A: Abnormal. * *P*_FDR_ < 0.05.

Fourteen infants with normal FMs had stiff movement character. When we compared infants having normal movement character (*N* = 9) with this group, we observed an increased rsFC between the left superior occipital gyrus and both the ipsilateral (Normal: 0.19 ± 0.03 vs. Stiff: 0.04 ± 0.06; *P*_FDR_ = 0.024) and contralateral (Normal: 0.11 ± 0.03 vs. Stiff: −0.08 ± 0.06; P_FDR_ = 0.03) posterior cingulate gyrus (Figure 5).

#### 3.3.3. Jerky Movement Character is Associated with Increased Connectivity between Visual Cortex and Cingulate Cortices

**Figure 6:**
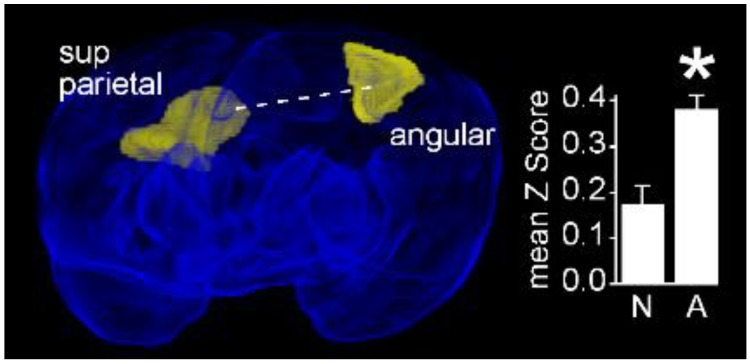
The left angular gyrus exhibits a decreased functional connectivity in infants with normal movement character compared with infants with jerky movement character. Obliquely projected 3D reconstructions of MRI images of cerebral grey matter and identified parcellated regions derived from a neonatal atlas. Dashed white line depicts the connectivity relationship. Inset: Fisher-transformed Z-scores ± 1 SEM. N: Normal; A: Abnormal. * *P*_FDR_ < 0.05.

Twenty-two infants with normal FMs had jerky movement character. When we compared infants having normal movement character (*N* = 9) with this group, we observed a decreased rsFC between the left angular gyrus and the contralateral superior parietal gyrus (Normal: 0.18 ± 0.06 vs. Jerky: 0.38 ± 0.03; *P*_FDR_ = 0.014. Figure 6).

#### 3.3.4. Tremulous Movement Characters is not Associated with Differences in Regional Connectivity

When we compared infants normal FMs and tremulous movement character (*N* = 11) with infants having normal movement character, we found no regions with statistically significant differences in connectivity between region pairs. 

### 3.4. Resting State Functional Connectivity Correlates of Normal and Adverse Long-Term Neurodevelopmental Outcomes

**Figure 7:**
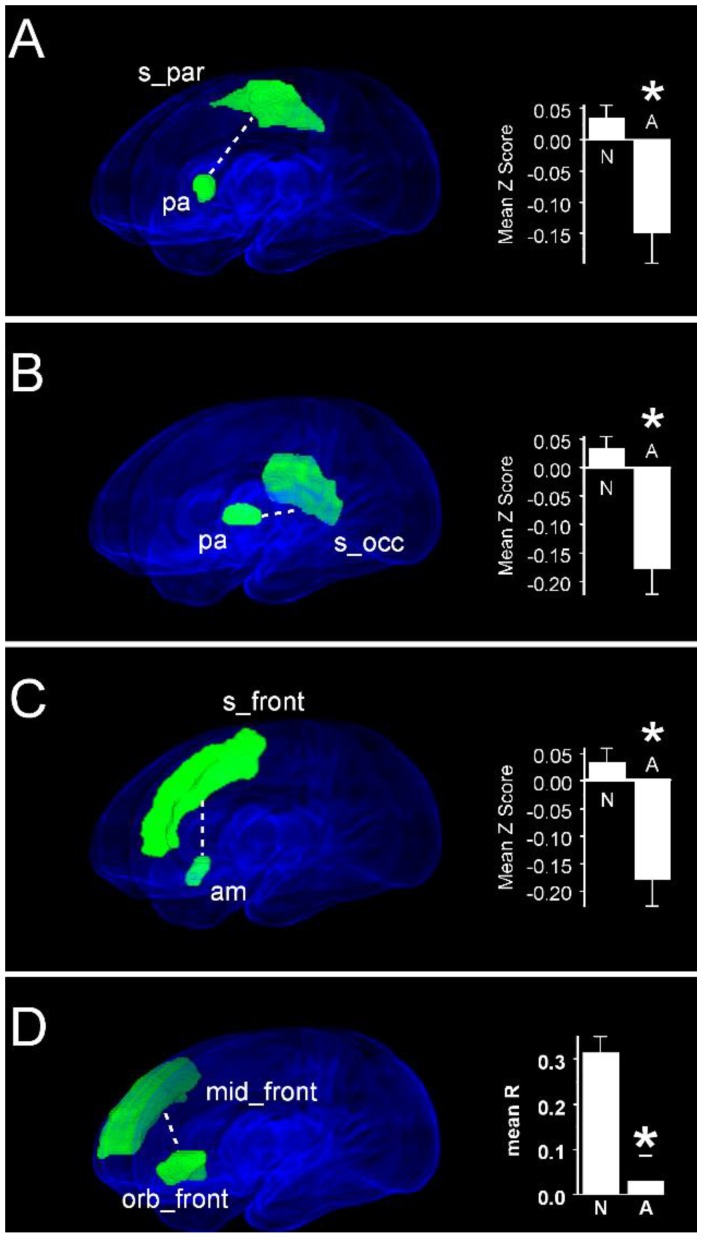
Region pairs exhibiting increased and decreased functional connectivities in infants with normal scores on the cognitive subscale of the Bayley III assessment at two years post-menstrual age compared with infants with scores <85. Obliquely projected 3D reconstructions of MRI images of cerebral grey matter and identified parcellated regions derived from a neonatal atlas. Dashed white lines depict connectivity relationships. Inset: Fisher-transformed Z-scores ± 1 SEM. (**A**) Pallidum and superior parietal gyrus. (**B**) Pallidum and superior occipital gyrus. (**C**) Amygdala and superior frontal gyrus. **(D**) Middle frontal cortex ad orbital frontal cortex. N: Normal; A: Abnormal. * *P*_FDR_ < 0.05.

We next compared functional connectivity strength between groups of infants with normal (score ≥ 85) and abnormal (score < 85) subscale-specific scores on Bayley III assessment at two years. Compared with children with abnormal cognitive scores, infants with normal cognitive scores exhibited increased functional connectivities between the pallida and vision-related parietal-occipital regions (Figure 7). These increased pallidal connectivities were between the right pallidum and ipsilateral superior parietal cortex (Normal: 0.04 ± 0.02 vs. Abnormal: −0.15 ± 0.05; *P*_FDR_ = 0.008, Figure 7A), and between the left pallidum and contralateral superior occipital cortex (Normal: 0.03 ± 0.02 vs. Abnormal: −0.18 ± 0.05; *P*_FDR_ = 0.003, Figure 7B). These infants also had greater connectivity between right amygdala and the contralateral superior frontal gyrus (Normal: 0.04 ± 0.02 vs. Abnormal: −0.18 ± 0.05; *P*_FDR_ = 0.017, Figure 7C). Finally, these infants had significantly greater functional connectivity between the left orbitofrontal gyrus and the right middle frontal gyrus (Normal: 0.32 ± 0.04 vs. Abnormal: −0.03 ± 0.06; *P*_FDR_ = 0.017, Figure 7D). Notably, we found no regions pairs between which functional connectivity was decreased in infants with normal compared with abnormal cognitive scores.

**Figure 8:**
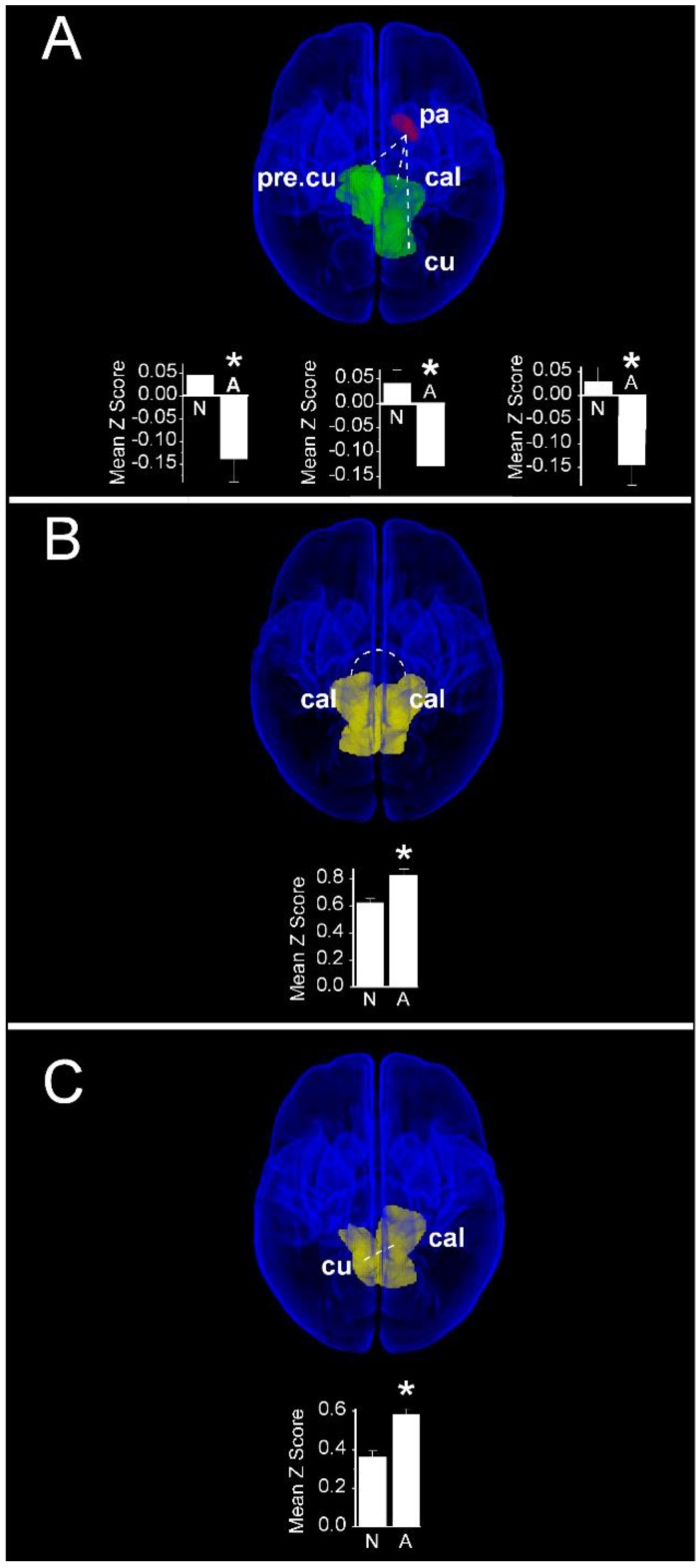
Region pairs exhibiting increased and decreased functional connectivities in infants with normal (≥85) and abnormal (<85) scores on the motor subscale of the Bayley III assessment at two years post-menstrual age. Obliquely projected 3D reconstructions of MRI images of cerebral grey matter and identified parcellated regions derived from a neonatal atlas. Dashed white lines depict connectivity relationships. Inset: Fisher-transformed Z-scores ± 1 SEM. (**A**) Increased connectivity between pallidum and visual cortex regions: left: left pallidum and contralateral precuneus; middle: Left pallidum and ipsilateral calcarine cortex; and right: Left pallidum and ipsilateral cuneus. (**B**) Decreased connectivity between bilateral calcarine cortices; (**C**) Decreased connectivity between left calcarine and right cuneus. N: Normal; A: Abnormal. * *P*_FDR_ < 0.05.

In contrast to cognitive scores, increased functional connectivity in infants with normal motor scores compared with those having abnormal motor scores were restricted to interactions between the left pallidum and vision-related cortical regions (Figure 8A). Compared with children with abnormal motor scores, infants with normal motor scores demonstrated a greater functional connectivity between the left pallidum and each of the right precuneus (Normal: 0.05 ± 0.03 vs. Abnormal: −0.14 ± 0.05; *P*_FDR_ = 0.015), left cuneus (Normal: 0.03 ± 0.03 vs. Abnormal: −0.14 ± 0.04; *P*_FDR_ = 0.015), and left calcarine cortex (Normal: 0.04 ± 0.03 vs Abnormal: −0.13 ± 0.04; *P*_FDR_ = 0.018). Notably, these infants also demonstrated a weaker functional connectivity compared with children having abnormal motor scores between bilateral primary visual (calcarine) cortices (Normal: 0.62 ± 0.03 vs. Abnormal: 0.83 ± 0.05; *P*_FDR_ = 0.018, Figure 8B), as well as between the left calcarine and the contralateral cuneus cortex (Normal: 0.36 ± 0.03 vs. Abnormal: 0.59 ± 0.05; *P*_FDR_ = 0.018, Figure 8C). Finally, we observed no significant differences in functional connectivity when infants with normal and abnormal language scores were compared. 

### 3.5. Functional Connectivity Increases as a Linear Function of Increasing Scores on Assessments of Long-Term Neurodevelopmental Outcomes

**Figure 9:**
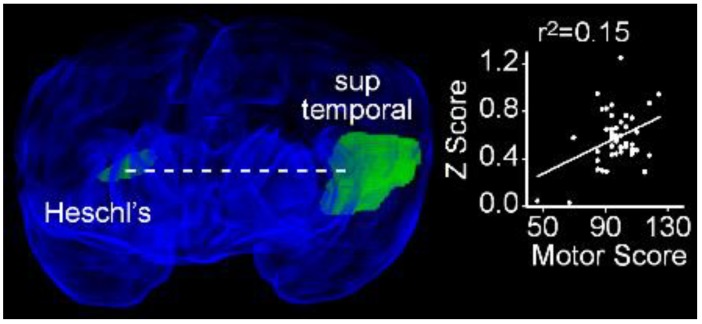
Resting state functional connectivity between the left superior temporal gyrus and contralateral Heschl’s gyrus exhibits a positive linear relationship with performance on the motor subscale of the Bayley III. Obliquely projected 3D reconstruction of MRI images of cerebral grey matter and identified parcellated regions derived from a neonatal atlas. Dashed white line depicts the connectivity relationship. Inset: Individual Fisher-transformed Z-scores of correlation coefficients between regions.

Having identified rsFC differences between normal and adverse long-term neurodevelopmental outcomes, we searched for brain regions between which functional connectivity at term demonstrated a significant linear relationship as a function of performance at two years on subscales of the Bayley III. We identified a single region pair in which rsFC increased as a function of performance on the motor subscale: the left superior temporal gyrus and the contralateral Heschl’s gyrus (r^2^ = 0.15; *P*_FDR_ = 0.016, Figure 9). 

**Figure 10:**
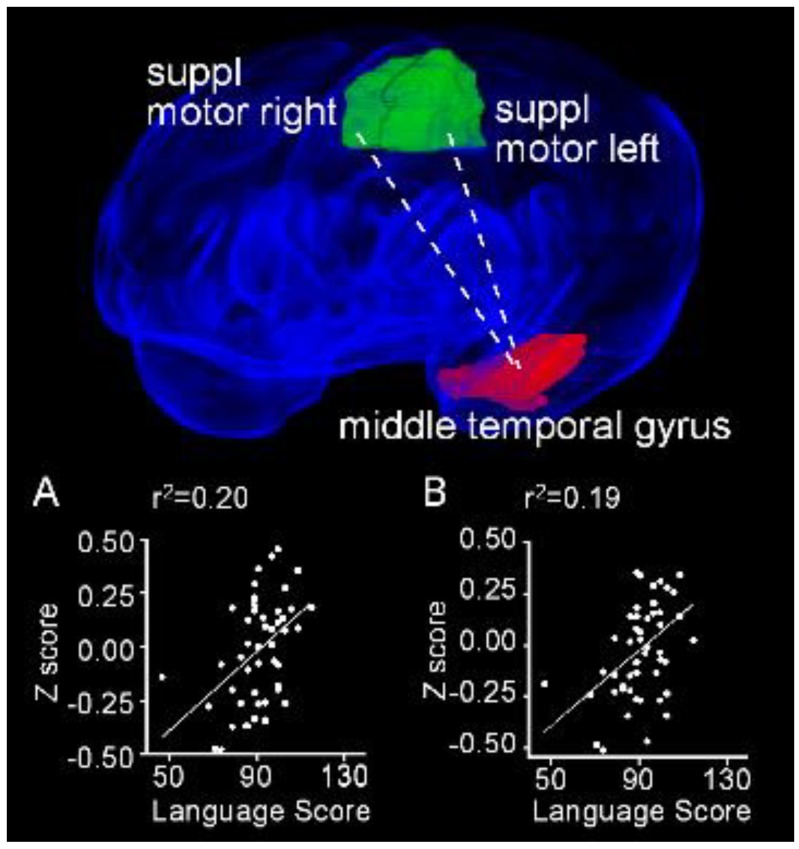
Resting state functional connectivity between the left middle temporal gyrus and both right and left supplementary motor areas exhibit positive linear relationships with performance on the language subscale of the Bayley III. Obliquely projected 3D reconstruction of the MRI images of cerebral grey matter and identified parcellated regions derived from a neonatal atlas. Dashed white line depicts the connectivity relationship. Inset: Individual Fisher-transformed Z scores of the correlation coefficients between regions. (**A**) Middle temporal gyrus and right supplemental motor area. (**B**) Middle temporal gyrus and left supplemental motor area.

We identified no region pairs exhibiting a significant linear relationship between rsFC and performance on the cognitive subscale. However, an analysis of performance on the language subscale of the Bayley III and rsFC did identify two region pairs that demonstrated significant positive linear relationships between scores and rsFC. Specifically, the rsFC between the left middle temporal gyrus and each of the ipsilateral (r^2^ = 0.20; *P*_FDR_ = 0.017) and contralateral (r^2^=0.19; *P*_FDR_ = 0.017) supplementary motor areas increased linearly as a function of language performance (Figure 10).

## 4. Discussion

In this study, we found that very premature infants who develop normal spontaneous movements at 12–15 weeks of age have significant differences in resting state connectivity between pairs of brain regions at term compared with infants who do not develop normal spontaneous movements. Importantly, we also found that, in these infants, those who go on to have normal motor or cognitive function at two years of age demonstrate significant differences in resting state connectivity at term between a different set of brain region pairs compared with infants with abnormal function. Importantly, the incidences of the major medical complications of prematurity that strongly affect neurodevelopmental outcomes, including bronchopulmonary dysplasia, severe intraventricular hemorrhage, and periventricular leukomalacia, and the treated retinopathy of prematurity did not differ between our infants with normal and aberrant FMs.

Our approach, which identified differences in regional resting state connectivity determined by subsequent test performance, contrasts with studies of resting state connectivity that have employed hypothesis-free, independent component analysis to identify and characterize resting state networks in term and preterm infants [12,13,14]. As reviewed recently [6,25], reduced connectivity, specifically within the default mode network, has been associated with prematurity [26,27]. Here, we similarly performed an exploratory, hypothesis-free study in which we correlated the time-dependent BOLD signal variation between each of the 90 brain regions with that of every other region. 

In this exploratory study, a 5% false positive threshold (*p* < 0.05) of statistical significance yielded a large number of false positives (405) arising from 8100 comparisons. To address this issue, we employed the seed-level false discovery rate [28] correction of our results. As a result, this approach focused on identifying regional functional connectivity in abnormally developing children that differs from those of typically developing children, and it may provide new insights into the abnormal functional brain development of premature infants.

Compared with infants with aberrant FMs, infants with normal FMs demonstrated increased resting state connectivity between the putamen and two associative cortical regions (inferior parietal and fusiform gyri) and between the inferior parietal gyrus and the amygdala. When we employed the more detailed, continuous MOS, we observed graded increases in resting state connectivity between these same region pairs. The similarity of the identified region pairs suggests that the additional skills evaluated by the MOS are mediated by the same regions that mediate the development of FMs. 

The increased functional connectivity we observed between the putamen and associative cortical regions in infants with normal FMs suggests that cortical inputs to the basal ganglia, a well described hub for the regulation of motor activity [29], is an important determinant of the development of FM. Interestingly, it has been suggested that the development of FM is a manifestation of the process of refining proprioception–motor integration during development [30]. Our finding that both the inferior parietal and fusiform gyri, brain regions responsible for the integration of sensory information, had greater connectivity with the basal ganglia in children with normal FMs compared to those with aberrant FM supports this concept. 

Using the MOS, we identified a superset of region pairs found in the FM analysis having a significantly increased resting state connectivity. Identifying these additional pairs, the magnitude of whose functional connectivity was linearly associated with MOS performance, is perhaps unsurprising, as the MOS quantifies the features of motor performance that are likely to be modulated by non-motor regions, such as smiling, visual scanning or excitement bursts. One of these region pairs—the angular and inferior frontal gyri—mediates aspects of cognition and auditory language comprehension in older children and adults [31,32], suggesting that motor behavior in infancy is related to subsequent language development. The observation that higher MOSs in infancy are associated with better expressive, but not receptive, language at four and ten years of age [7] supports this idea.

Our resting state connectivity analyses differentiated infants who went on to score in the normal range on Bayley III scales of cognitive or motor development at two years of age from those who do not. Compared with infants with abnormal Bayley III motor scores, infants with normal scores exhibited increased resting state connectivity between pallidum and bilateral primary visual and associative visual cortices. At the same time, functional connectivity between individual components of the visual cortex (bilateral calcarine cortices, calcarine–contralateral cuneus) were significantly decreased (Figure 6B). The increased connectivity between pallidum and visual cortex that we saw in infants with intact cognition parallels the positive association between cognition at two years of age and the degree of structural connectivity between thalamus and cortical regions in preterm infants [33]. In addition, the improved cortical–subcortical connectivity reported in 12 year-olds who were born at term compared with those who were born preterm [34] provides additional support for the concept that increased cortical–subcortical connectivity is critical for motor development through childhood.

In infants with normal Bayley III cognitive scores, the increased connectivity between pallidum and visuospatial associative cortices (superior parietal and superior occipital gyri) compared with infants having abnormal scores suggest analogous conclusions regarding the role of basal ganglia–visuospatial integration in developing cognitive skills in infancy. While it is not immediately clear how increased connectivity between the amygdala and contralateral superior frontal cortex may be interpreted, the positive association between amygdala functional connectivity and Intelligence Quotient at four years of age [35] suggests that our detection of this region pair as functionally connected is not by chance. The identification of region pairs with significant differences in resting state connectivity as a function of neurodevelopmental outcomes indicates that studies of resting state connectivity in infants can provide insights into the mechanisms underlying normal and abnormal brain development. Finally, decreases in regional resting state connectivity in children with normal neurodevelopmental outcomes may indicate that children with abnormal outcomes are delayed in the normal decline of regional connectivity.

Surprisingly, as children’s scores improved on the motor subscale of the Bayley III, we found a positive linear association with connectivity between the left superior temporal gyrus and the contralateral Heschl’s gyrus (transverse temporal gyrus)—regions subsequently participating in language recognition [36]. Notably, we also found that, as language performance on the Bayley III improved, so too did connectivity between the middle temporal gyrus, a component of the ventral stream of auditory language processing [36], and the supplementary motor cortices, regions also associated with speech and language comprehension [37]. While the roles played by these specific cortical regions in at term-equivalent age may be difficult to interpret, these associations may provide early functional biomarkers of neurodevelopmental trajectory and outcome. While a group of experts has recommended that very preterm infants be screened with both structural MRI at term and general movement assessments at 10–15 weeks of corrected age [38], fMRI assessment has not yet been extended to the clinical evaluation of individual infants. The current absence of understanding of the range of normal resting state functional connectivity among infants precludes the use of fMRI to assess and grade individual infants for clinical prognostication, and it requires further studies.

The MOS, which includes MR and FM scores, improved the ability of motor behavior assessment at three months of age to account for cognitive, language, and motor Bayley III scores at two years of age. This improvement suggests that the MR score provides information that does not overlap with information provided by an FM assessment. The improvement also suggests that the behaviors assessed by the MR score reflect brain function important for long-term motor and non-motor neurodevelopment. Our observation that much of the variance in MR score is accounted for the presence of BPD demonstrates the importance of BPD in inhibiting motor development. Indeed, in multiple regression analyses of very low birth-weight infants, controlled for socioeconomic class and neurologic complications of prematurity, the presence of BPD has been reported to have an independent negative effect on motor outcomes. Multiple studies have since reported strong independent associations between BPD and abnormal neurodevelopmental outcomes, including cerebral palsy [39,40], gross and fine motor skills [41], and neurocognitive delays [42,43,44]. Together, these strong associations suggest that BPD induces deficits in brain regions mediating long-term neurodevelopment, perhaps as a result of the recurrent hypoxia suffered during the newborn period [45].

Specific components of the MOS at three months post-term age have been associated with neurocognitive development in very low birthweight infants: monotonous, jerky, or stiff movement characters at three months of age have been associated with cognitive abnormalities at school age [46], as have abnormal postural patterns [47]. The increased connectivity between the somatosensory cortex and several visuospatial association cortices we identified in children without monotonous movement character suggests that decreased interactions between these region pairs provides biomarkers for infants who are at risk for cognitive problems. Similarly, infants having stiff movement character exhibited a decreased connectivity in brain region pairs (posterior cingulate and superior occipital) subsequently mediating attention and visual processing [48]. The finding that children with abnormal movement character have significantly higher reports of inattention at age 10 [49] compared with children with normal movements, suggests that the early interaction between these brain regions may indicate a risk for later attentional deficits. Similar conclusions may be drawn for the regions that we identified as having significantly decreased connectivity in infants without jerky movement character. 

## 5. Conclusions

The identification of specific pairs of brain regions between which resting state functional connectivity at term equivalence is associated with abnormal motor behavior at three months of age provides candidate brain regions that contribute to the development of a normal infant motor repertoire. Importantly, the identification of similar region pairs that are associated with abnormal long-term neurodevelopmental cognitive and motor outcomes provides insight into the mechanisms underlying normal and abnormal infant development as a whole. Understanding how functional connectivity differs between rest and during fidgety movements themselves at three months of age would definitively identify regions that contribute to these movements. However, the technical difficulties in obtaining true, task-specific fMRI scanning in infants remain challenging. 

## Figures and Tables

**Table 1:** Demographic and clinical characteristics of the study cohort.

Characteristic	*N* = 123
Male (*n* (%))	68 (55.3)
Gestational Age (weeks, mean ± SD)	27.1 ± 2.0
Gestational Age < 28 weeks (*n* (%))	67 (54.9)
Birth Weight (grams, mean ± SD))	973 ± 269
Birth weight < 1000 g (*n* (%))	63 (51.6)
Oxygen support >36 weeks (*n* (%))	42 (34.7)
Necrotizing enterocolitis * (*n* (%))	10 (8.3)
Treated retinopathy of prematurity (*n* (%))	14 (11.6)
Severe intraventricular hemorrhage † (IVH; *n* (%))	9 (7.4)
Periventricular leukomalacia (PVL; *n* (%))	6 (5.0)
Severe IVH or PVL (*n* (%))	12 (9.8)
Normal FMs rating (*n* (%))	101 (82.8)
Motor Optimality Score (mean ± SD)	21.3 ± 6.3
Cerebral Palsy (*n* (%))	10 (8.3)
Bayley III Cognitive (mean ± SD)	90.8 ± 17.3
Bayley III Language (mean ± SD)	88.4 ± 14.5
Bayley III Motor (mean ± SD)	92.5 ± 15.5

* Bells stage IIIB or greater; † Papile grade III or IV.

**Table 2:** Comparison of the extent to which the fidgety movement (FM) score or the motor optimality score (FM plus motor repertoire (MR) scores) better accounts for the variance in infants’ scores on Bayley III subscales of infant development, using linear regression models. Values are the beta coefficients of the independent variables of the regression equations with 95% confidence intervals in parentheses.

	Cognitive		Language		Motor	
	Model 1 (FM only)	Model 2 (FM + MR)	Model 1 (FM only)	Model 2 (FM + MR)	Model 1 (FM only)	Model 2 (FM + MR)
FM coefficient	1.43 (0.63–2.24) *p* < 0.001	0.86 (0.04–1.68) *p* = 0.04	1.21 (0.55–1.87) *p* < 0.001	0.83 (0.14–1.51) *p* = 0.02	1.33 (0.63–2.04) *p* < 0.001	0.79 (0.07–1.50) *p* = 0.03
MR coefficient	-	2.04 (0.95–3.12) *p* < 0.001	-	1.37 (0.46–2.29) *p* = 0.004	-	1.96 (1.01–2.91) *p* < 0.001
R-squared	0.11	0.20	0.11	0.16	0.12	0.23

**Table 3:** Statistical comparison of clinical characteristics likely to affect neurologic functioning in infants who had functional MRI scans and General Movement assessments with those who had General Movement assessments only.

	Scanned (*N* = 47)	Not Scanned (*N* = 70)	*P* (Unadjusted)
Male (*N*, %)	27 (57.4)	41 (54.0)	0.71
Mean Gestational age (weeks, ± SD)	27.2 ± 0.3	27.0 ± 0.2	0.50
Mean Birth Weight (g ± SD)	994 ± 28	961 ± 32	0.63
Bronchopulmonary dysplasia * (*N*, %)	11 (24.4)	31 (40.8)	0.08
Treated retinopathy of prematurity (*N*, %)	3 (6.7)	11 (14.5)	0.25
Severe IVH or PVL (*N*, %)	4 (9.5)	8 (10.5)	1.00

* supplemental oxygen after 36 wks post conceptional age.

**Table 4:** Comparison of clinical characteristics of scanned infants with normal and aberrant FMs.

	Normal FMs (*N* = 41)	Aberrant FMs (*N* = 12)	*P*
Male	26 (63.4)	4 (33.3)	0.10
Mean Gestational age (weeks, ± SD)	27.6 ± 1.6	26.6 ± 2.0	0.07
Mean Birth Weight (g, ± SD)	1050 ± 222	756 ± 217	0.001
Mean Post-term age at GMA testing (weeks, ± SD)	12.0 ± 1.3	11.92 ± 1.5	0.85
Bronchopulmonary dysplasia * (*N*, %)	12 (28.95)	2 (66.7)	0.48
Treated retinopathy of prematurity (*N*, %)	3 (5.26)	2 (33.3)	0.31
Severe IVH or PVL (*N*, %)	2 (4.9)	3 (25)	0.07
Mean Post-conceptional age at MRI (weeks, ± SD)	38.99 ± 2.52	37.61 ± 3.04	0.16

* supplemental oxygen after 36 weeks post conceptional age. GMA, General Movement Assessment.
